# Natural Therapeutics in Aid of Treating Alzheimer’s Disease: A Green Gateway Toward Ending Quest for Treating Neurological Disorders

**DOI:** 10.3389/fnins.2022.884345

**Published:** 2022-05-16

**Authors:** Basharat Ahmad Bhat, Abdullah Almilaibary, Rakeeb Ahmad Mir, Badr M. Aljarallah, Wajahat R. Mir, Fuzail Ahmad, Manzoor Ahmad Mir

**Affiliations:** ^1^Department of Bioresources, School of Biological Sciences, University of Kashmir, Srinagar, India; ^2^Department of Family and Community Medicine, Faculty of Medicine, Albaha University Alaqiq, Alaqiq, Saudi Arabia; ^3^Department of Biotechnology, Baba Ghulam Shah Badshah University, Rajouri, India; ^4^Department of Gastroenterology and Hepatology, Qassim University, Buraydah, Saudi Arabia; ^5^College of Applied Medical Science, Majmaah University, Al Majma’ah, Saudi Arabia

**Keywords:** Alzheimer’s disease, phytoconstituents, inflammation, neurological disorders, effective treatments

## Abstract

The current scientific community is facing a daunting challenge to unravel reliable natural compounds with realistic potential to treat neurological disorders such as Alzheimer’s disease (AD). The reported compounds/drugs mostly synthetic deemed the reliability and therapeutic potential largely due to their complexity and off-target issues. The natural products from nutraceutical compounds emerge as viable preventive therapeutics to fill the huge gap in treating neurological disorders. Considering that Alzheimer’s disease is a multifactorial disease, natural compounds offer the advantage of a multitarget approach, tagging different molecular sites in the human brain, as compared with the single-target activity of most of the drugs so far used to treat Alzheimer’s disease. A wide range of plant extracts and phytochemicals reported to possess the therapeutic potential to Alzheimer’s disease includes curcumin, resveratrol, epigallocatechin-3-gallate, morin, delphinidins, quercetin, luteolin, oleocanthal, and other phytochemicals such as huperzine A, limonoids, and azaphilones. Reported targets of these natural compounds include inhibition of acetylcholinesterase, amyloid senile plaques, oxidation products, inflammatory pathways, specific brain receptors, etc. We tenaciously aimed to review the in-depth potential of natural products and their therapeutic applications against Alzheimer’s disease, with a special focus on a diversity of medicinal plants and phytocompounds and their mechanism of action against Alzheimer’s disease pathologies. We strongly believe that the medicinal plants and phytoconstituents alone or in combination with other compounds would be effective treatments against Alzheimer’s disease with lesser side effects as compared to currently available treatments.

## Graphical Abstract

The graphical abstract displays therapeutic potential of plant extracts and their derived metabolites such as curcumin, resveratrol, epigallocatechin-3-gallate, huperzine A, limonoids, and azaphilones etc., to reduce the clinical features associated with Alzheimer’s disease and possible end to quest for global prevalence of AD in humans.

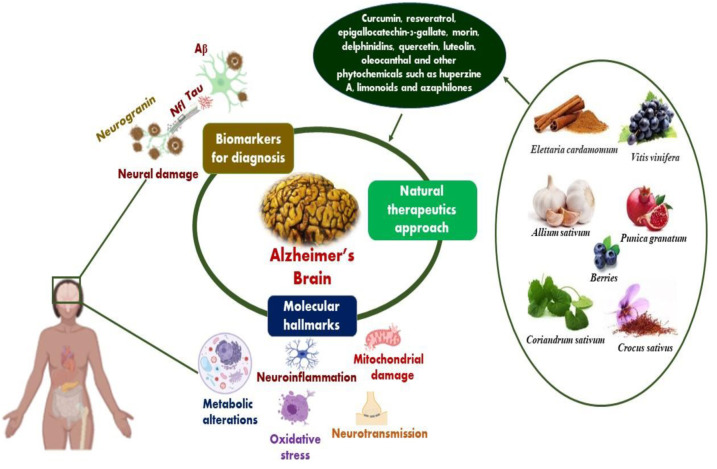

## Introduction

**FIGURE 1:**
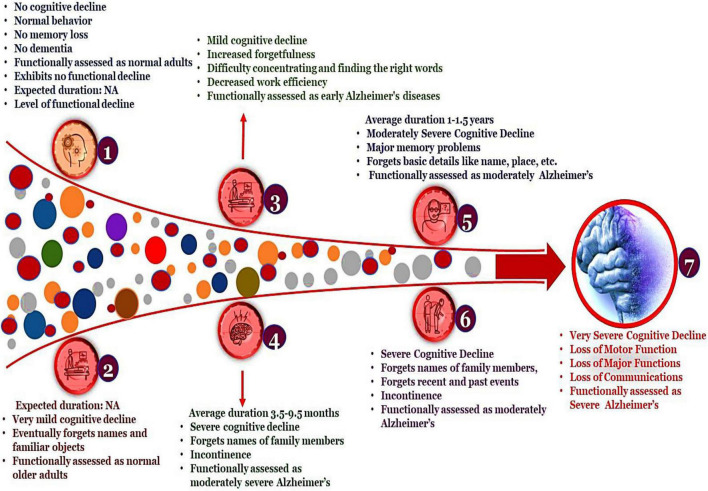
Different stages of Alzheimer’s disease. The above classification is based on the Global Deterioration Scale/Reisberg Scale for rating dementia. This classification of AD mainly relies on the Functional Assessment Staging Test (FAST) wherein the individual is examined for their cognitive decline depending on their aptness to engage in daily activities.

Alzheimer’s disease (AD) is attributed to the inception of amyloid plaques and tangled fibers which consequently results in neurodegeneration featured by impairment of cognitive function and amnesia (memory loss) (Anand et al., 2014; Sahebkar et al., 2021). AD manifests the highest prevalence in the elderly and is adjudged as predominant neurodegenerative disorders, ostensive with limited and inefficacious treatment regimes. Discerning the pathophysiology expounds the significant hallmarks of AD and assists in diagnosis wherein a patient is screened for one or more of the following characteristics: amnesia (memory loss), aphasia (expressive aphasia is an inability to find right words while receptive aphasia demonstrates an inability to understand), apraxia (loss of motor function) and agnosia (loss of functioning of 5 senses) (Sahebkar et al., 2021). The FDA (Food and Drug Association) approved drugs for the treatment of AD includes the administration of AChEIs (acetylcholinesterases inhibitors), NMDA (N-methyl-D-aspartate receptor antagonists) (Auld et al., 2002; Anand et al., 2014), Selegiline (used in the treatment of Parkinson’s Disorder) (Anand et al., 2014; Abeysinghe et al., 2020), estrogen therapy (Auld et al., 2002), NSAIDs (Non-Steroids Anti-Inflammatory Drugs) (Abeysinghe et al., 2020). A comprehensive overview of these drugs is listed in Table 1. Addressing Alzheimer’s is not limited to timely diagnosis and implementation of constructive treatment plans. The preponderance of AD in the elderly is often misleading as the symptoms are misread for aging. The classification of different stages of the disease progression of AD is depicted in Figure 1. Proper intervention in accordance with diseases progression ameliorates disease management. The irreversible damage to the brain cells and involuted pathophysiological, events associated with AD have always emphasized the need for the development of novel drugs and therapeutics, which render better outcomes with fewer or no side effects.

**TABLE 1:** FDA approved drugs in the treatment of Alzheimer’s diseases.

Generic name	Target	Type	Treated for	Function	Possible side effects
Aducanumab Aduhelm	Beta-amyloid	anti-amyloid antibody intravenous (iv) infusion	Alzheimer’s disease	Enhances Memory, orientation language	Abnormal brain changes Headache Swelling in the brain
**Cholinesterase inhibitors**					
Donepezil Aricept	Acetylcholine esterase	Oral drug	All stages of Alzheimer’s disease	Cholinergic transmission Increases synaptic availability of acetylcholine	Nausea Vomiting loss of appetite increased frequency of bowel movements. Stomach pain Weight loss
Rivastigmine Axelon	Acetylcholine esterase	Oral or transdermal patch	Mild-moderate Alzheimer’s disease	Treats dementia	Nausea Vomiting loss of appetite o Stomach pain Weight loss
Galantamine Razadyne	Acetylcholine esterase	Oral drug	Mild-moderate Alzheimer’s disease	Improves the function of nerve cells in the brain	Nausea Vomiting Diarrhea Heartburn Headache Pale skin
Memantine Namenda	NMDA receptor antagonist	Oral drug	Moderate- severe Alzheimer’s disease	Increases normal brain functioning, memory, cognition	Headache Constipation Sleepiness Dizziness Aggression
Memantine + donepezil Namzaric	NMDA receptors	Oral drug	Moderate- severe Alzheimer’s disease	Restores neurotransmitters	Cramps Nausea Convulsions Difficulty in urinating
Suvorexant (Belsomra)	Orexin receptor antagonist	Oral drug	Mild-moderate Alzheimer’s disease	Improves behavior and psychological symptoms	Drowsiness Dizziness Headache Cough Diarrhea

The plethora of bioactive phytocompounds, useful vitamins and chemicals has maximized the need to derive their therapeutic potentials (Garriga et al., 2015). Their feasibility to be taken as a dietary supplement, unparallel chemical diversity, remarkable efficacy, and their dexterity to interact with biological targets to positively revamp biological functions is superlative (Mir and Albaradie, 2014; Yuan et al., 2016). The presence of alkaloids, flavonoids, carotenoids, and other phytonutrients in natural products is the underpinning for nutraceuticals, which believes that diet has an undeniable effect on epigenetics (Mir and Agrewala, 2008; Mir, 2015). Moreover, the consumption of functional foods which enhances brain functioning (often termed as brain foods) will aid in overall management of AD (Mir and Albaradie, 2015; Bhat et al., 2022). Though the current research is oriented toward food-based novel drugs, it is fundamental to develop pharmacological preparations to address AD. The principle of nutraceuticals emphasizes developing interventions that are beyond the diet and healthy eating and shouldn’t be misapprehended for devising a diet plan rich with supplements as this can only compensate the nutritional requirements and is ineffective to combat a malady. Significant pharmacological properties like neuroprotective, anti-oxidant, anti-inflammatory, anti-apoptotic, etc., demonstrated by phytonutrients like tannins, alkaloids, phenols, carotenoids can be inspected to devise potential drugs (Mir et al., 2019; Lautie et al., 2020; Qadri et al., 2021). In this review, we will constructively look into important hallmarks of AD, neuroprotective and anti-Alzheimer’s properties exhibited by phytonutrients and explore their food sources.

## Pathophysiology Associated With Alzheimer’s Diseases

The prominent hallmarks of AD are hypothesized to be the production of Aβ plaques and the neurofibrillary tangles (NFTs) in different regions of patients. Further, AD is grossly progressed by aberrant phosphorylation and agglomeration of neurofibrillary tau proteins (Majeed et al., 2021), causing instability of microtubules and concomitantly includes functional abnormalities in the axon transportation (Atlante et al., 2020).

**FIGURE 2:**
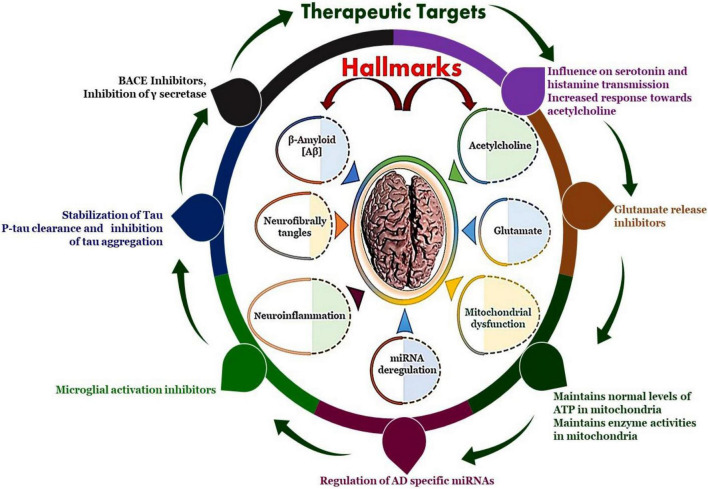
The neuropathological hallmarks of Alzheimer’s disease: formation of amyloid-beta plaques, formation of neurofibrillary tangles, miRNA deregulation, mitochondrial dysfunction, neuroinflammation and therapeutic targets such as BACE inhibitors and Inhibition of γ secretase, stabilization of Tau protein, influence on serotonin and histamine transmission, maintains normal levels of ATP in mitochondria and maintains enzyme activities in mitochondria, microglial activation inhibitors and regulation of specific miRNAs.

Subsequently the cognitive impairment and neurodegeneration is currently thought to be a primary driver for NFTs (Dey et al., 2017). These occurrences suggest a link between aberrant tau proteins and memory deficits in Alzheimer’s patients associated with various AD associated hallmarks and recognized potential therapeutic targets (Figure 2). Originally, it was presumed that Aβ peptide accumulation caused abnormal modifications of tau functioning, these processes work in tandem, amplifying each other’s detrimental consequences and causing the intellectual loss associated with AD (Mir et al., 2018; Mir). The evolved Aβ are further deposited in hippocampus and basal segment to form amyloid plaques and recruits the Aβ insoluble aggregates and hence damage to mitochondria resulting in severe decline in production of ATPs (Mir et al., 2021). Subsequently the astrocytes and microglia induce oxidation and inflammatory related reactions after activation leading to dysfunctioning of neurons and their apoptosis to lead clinical features of Alzheimer’s disease. Additionally, Aβ activate tau protein kinase which in turn phosphorylates the tau proteins (Shao and Xiao, 2013).

**FIGURE 3:**
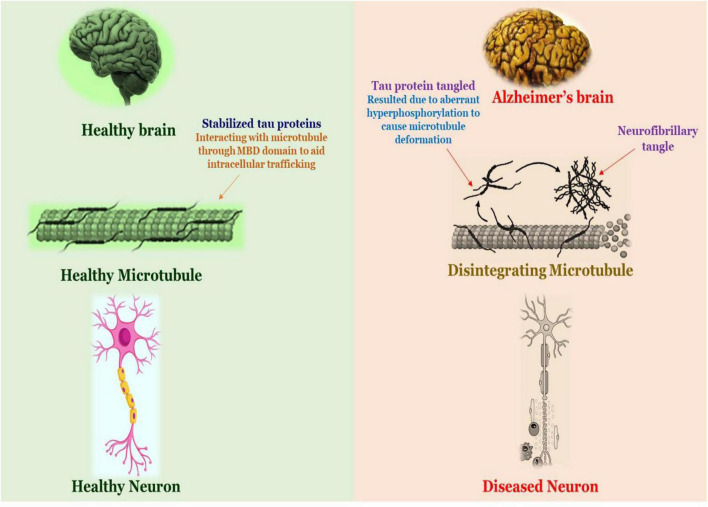
Schematic diagram of the pathology of Alzheimer’s disease.

Following an extensive investigation into the processes of Aβ peptide-related damage, the underlying mechanisms that induce toxicity remain unknown. Researchers have indicated that Aβ aggregate receptor interaction influences several critical neuronal processes, but they haven’t disclosed the whole profile of these receptors or the associated signal transduction pathways linked with them, implying that further investigation is necessary (Yadav, 2021). The complexity of neurodegeneration is not fully decoded and therefore we have various hypothesis that attempts to decipher the pathophysiology of AD. The pathogenesis of Alzheimer’s disease is shown in Figure 3.

## The Cholinergic Hypothesis

Cholinergic synapses are omnipresent in the brain, nerve cells, and spinal cord. This synaptic conduction is essential for cognition, concentration, memory, attention, and other superior cognitive mental abilities. Multiple studies imply that cholinergic synaptic transmission plays a key role in enhancing cognitive performance, brain functioning, and plasticity. As a result, subsequent research is oriented toward investigating the normal cognitive abilities and age-associated cognitive impairments caused due to the brain’s cholinergic system (Kaur et al., 2021; Pluta et al., 2021). The cholinergic theory transformed Alzheimer’s investigational studies from an observational and explanatory neuropathological study to the present paradigm of synaptic neurotransmission. The identification of rapidly depleting pre-synaptic cholinergic markers in the cerebral cortex (Scheltens et al., 2016), validation of Nucleus Basalis of Meynert (NBM) as the provenance of cortical innervation and the affirmation of its neurodegeneration specific to AD (Fu et al., 2017). Further, corroborating the cognitive decline caused due to cholinergic antagonists are the breakthrough discoveries supporting this hypothesis (Kaur et al., 2021). According to researchers, cholinergic synaptic transmission (CST) is important to cultivate memory as well as establishing learning abilities and any dysfunction in this system results in cognitive decline. In AD, neurodegeneration in the basal forebrain and the hippocampus region is substantial (Bennett et al., 2017). The acetylcholine (ACh) produced during CST is hydrolyzed by acetylcholinesterase AChE and butyrylcholinesterase (BuChE) to terminate the signal conduction. The BuChE levels are either elevated or unaltered in AD patients. AChE agglomeration promotes the AD neurotoxic A fibrils, as AChE is fundamental for the production of neurotoxin A fibrils (Bloom, 2014).

## Tau Hypothesis

The pathophysiology of tau is initiated in the human brain even before the development of Aβ plaques, specifically aspiring the glutamate projection neurons. The aberrant phosphorylation of the tau among susceptible nerves in elderly rhesus macaques is linked to calcium imbalance (Hunt and Castillo, 2012; Vergara et al., 2019). The endosome that incorporates APP (Amyloid Precursor Protein) are confined by the improperly phosphorylated tau (pTau) on the microtubules, which eventually enhance Aβ synthesis. The aberrant tau phosphorylation caused by Aβ oligomers contributed to the pathogenesis of AD (Ferreira et al., 2015). Tau is a phosphoprotein composed of a microtubule-binding domain (MBD) and a projection domain in the brain, it entails 38 phosphorylation sites and has 6 isoforms composed of 352–441 amino acids. Additionally, the projection domain is separated into the proline-rich residual region and amino-terminal region. The tubulin-binding and carboxy-terminal regions encompass the MBD. Tau protein that has phosphorylated interfaces with tubulins to reinforce the assemblage of microtubules at the axon and is implicated in intracellular trafficking (Hampel et al., 2019). Normal tau is modified to paired helical filament tau (PHF-tau) and neurofibrillary tangles as a consequence of aberrant tau phosphorylation (NFTs). Hyperphosphorylated tau drastically alters microtubules and causes their deformation, ultimately culminating in the death of nerve cells. The proportion of hyperphosphorylated tau in an AD patient’s brain was 3–4 times greater than in a healthy brain (Hampel et al., 2018).

## Amyloid Hypothesis

The identification of amyloid-β as the principal constituent of senile plaques and tau protein was proved to be a cardinal element of the NFTs was a major milestone in AD research as it provides significant insights about the pathophysiology of AD. This was followed by the discovery of genetic variations in the APP which directed the research toward autosomal dominant familial Alzheimer’s disease. These findings, coupled with other research findings, observations, and assumptions have fostered the amyloid hypothesis, which posits that amyloid-beta is the principal component that facilitates the pathogenesis of Alzheimer’s disease (Bowen et al., 1976; Whitehouse et al., 1981). The discrepancy in the rate of Aβ42 and related Aβ proteins synthesis and elimination is the driving factor to initiate AD. APP695, APP751, and APP770 are the three isoforms of APP that are indispensable for neurogenesis, synaptic plasticity regulation, cell attachment, and intracellular stabilization of calcium ion levels. APP (soluble) exerts neurotrophic and neuroprotective effects (Kilimann et al., 2014). The primary amyloidogenic pathway generates soluble βA protein and C-terminal α residues by separating the Lys16 residue from APP utilizing the enzyme α-secretase. The non-amyloidogenic peptide p3 is then produced by the degradation of the C-terminal component α by the enzyme γ-secretase. When β-secretase degrades APP, it creates soluble βA peptides and the C-terminal β residues.

The latter is fragmented at numerous locations by the catalysis of γ-secretase, yielding in βA monomers, which contain 38–43 amino acid residues. Self-assemblage of the βA monomers into neurotoxic oligomers, proceeded by the development of fibrillary clusters, induces neural impairment, which eventually leads to dementia. The clustered oligomers also stimulate the production of amyloid plaques, which are a hallmark of AD. In AD patients, concentrations of the βA42 peptides are seen to be higher. The primary form of APP in the brain is a 695 amino acid membrane protein that is systematically broken by 2 specific enzymes amyloidogenic pathway, i.e., the β-site APP cleavage enzyme (BACE) and γ-secretase. It can be concluded that the underlying mechanism implicated in AD pathogenesis is the generation of insoluble βA peptides via disintegration of APP (Jiang et al., 2017). Further, current aim of therapeutic strategies is to overcome the global AD prevalence is to decrease the formation and subsequent aggregation of Aβ and their clearance from AD patients (Jakob-Roetne and Jacobsen, 2009; Morris et al., 2018; Barthélemy et al., 2019; Arnsten et al., 2021).

## Neuroinflammation

Anti-amyloid approaches were employed in the past to treat AD, but the outcomes were unsatisfactory. As demonstrated by autopsy and imaging experiments, the amyloid cascade hypothesis isn’t adequate to describe neural destruction in AD. Neuroinflammation has a key part in Alzheimer’s disease, however, the exact influence of neuroinflammation, presumably beneficial or detrimental, is still being questioned (Savelieff et al., 2018). The advent of neuroinflammation has been corroborated by investigations that reveal tissues of CNS has a sophisticated and naturally adaptable capacity to modify its fundamental paracrine systems through independently created and modulated inflammatory chemicals (Gallardo and Holtzman, 2019).

Neuroinflammation has distinct traits based on fundamental reasons, such as its persistence, intensity and severity of occurrence, and duration time. Age-associated deterioration of anti-inflammatory pathways generates inflammation and develops mild clinical signs, such as neural inflammatory responses followed by severe brain damage, which can persevere for few years before its clinical presentation as AD (Hampel et al., 2018). Overexpression of microglial cells and astrocytes promotes prolonged and recurrent neuroinflammation by releasing proinflammatory cytokines such as interleukins, TNF-α, and γ-interferon, which influence the central nervous system are detected in AD patients. The γ-secretase activity cleaves APP to generate βA peptides, which are stimulated by reactive oxygen species (ROS). The anti-inflammatory methodologies are implemented to produce new compounds to address AD (Barage and Sonawane, 2015).

## Oxidative Stress

The brain is highly susceptible to oxidative damage in comparison to other organs, as the constituents of nerve cells are oxidized in Alzheimer’s due to alterations in the functioning of mitochondria, which subsequently elevates the level of metal ions, inflammatory molecules, and β-amyloid (Aβ) proteins (Villaflores et al., 2012). Oxidative stress exerts a critical role in the development of AD. An accelerating accumulation of tau hyperphosphorylation, and consequent decrease of synaptic connections and neuronal cell, oxidative damage accelerates the pathogenesis of AD. Various experiments have validated the irreversible and severe damage of nerve cells caused due to oxidative stress (Lin et al., 2011). AD is a condition of aberrant aging and exhibits oxidative injury at concentrations that far exceed those of senior individuals (controls), implying the presence of other unknown factors that could have positively contributed to the pathogenesis of the disorder (Lin et al., 2011; Villaflores et al., 2012). The degradation of synaptic plasticity in the afflicted areas of the brain is thought to be the initial factor that prefaces neurodegeneration in AD, and it is linked to cognitive dysfunction. Oxidative stress advances the process of aging and the development of a plethora of neurological illnesses, notably Alzheimer’s disease. Excessive generation of reactive oxygen species (ROS) is linked to age/disorder-related mitochondrial dysfunction, disrupted mental equilibrium, and diminished antioxidant defense, which influences synaptic plasticity and neurotransmission, which directs toward cognitive impairment (Selkoe, 1991).

Reactive oxygen species also modulates DNA, triglycerides, peptides, amino acids, calcium levels, functioning efficacy and kinetics of mitochondrial, cellular morphology, receptors traffic, and energy balance, among other molecular targets (Hardy and Higgins, 1992).

## Natural Product-Based Therapeutics for Alzheimer’s Disease

The reported preventative properties of natural products are resultant of anti-oxidative or anti-neuroinflammatory effects, which action by blocking the agglomeration of Aβ and tau peptides and boosting cholinergic signaling. Natural compounds that tackle several pathogenic pathways may be capable of reducing/delaying or even preventing the occurrence and advancement of Alzheimer’s disease (Graham et al., 2017). Owing to the unavailability of efficacious pharmacological interventions for AD, alternative initiatives centered on diet modifications, intake of food supplements, consumption of functional food ingredients and organic products to avoid the disease conditions (Karran et al., 2011). Novel medications are critical for enhancing the quality of care and alleviating the symptoms of affected individuals. Multiple pharmacological options to diagnose and control Alzheimer’s disease are presented in recent times, but none have yielded optimistic results in clinical studies.

Recent studies have revealed that some dietary factors lower the incidence of AD, which has motivated scientists to investigate the benefits of phytoconstituents and extracted bioactive elements (Hensley, 2010; Calsolaro and Edison, 2016).

Natural remedies, have potential drug-like qualities, that enable them to pass the biological membranes, and allow them to interfere in protein-protein associations (Chen and Zhong, 2014; Wang X. et al., 2014; Calsolaro and Edison, 2016). Chemicals derived from different plant parts, including the roots, bulbs, tubers, rhizome, foliage, pods, seeds, and buds inhibit harmful amyloid plaque development and increase cholinergic signaling (Tönnies and Trushina, 2017). Antioxidant-rich foods have been shown to lower oxidative damage in the CNS. As a result, natural compounds have a wide spectrum of pharmacological effects, drawing the interest of scientists who want to use them in the production of therapeutic molecules to cure a variety of ailments (Kennedy and Wightman, 2011; Grant, 2016; Bhat et al., 2021; Chen X. et al., 2021).

**FIGURE 4:**
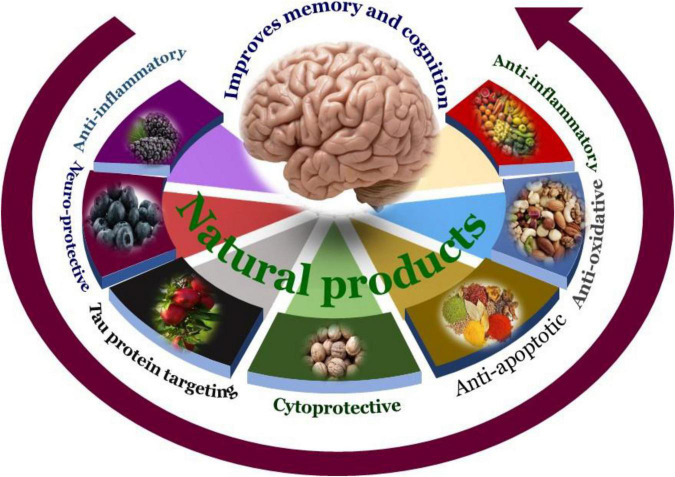
Overview of the natural products’ targets in AD. Many natural products show neuroprotective effects in the various experimental models of AD through multiple mechanisms of action. These include direct effect on neurotoxic agents such as Aβ plaque formation or tau hyperphosphorylation events.

The use of food and other natural sources as supplements to address several conditions is a traditional knowledge used in Ayurveda, Siddha, Unani, Chinese Herbal Medicines, and others in conventional traditional practices. Over the decades, the approach of utilizing these bioactive phytocompounds is changing constantly. Previously, the research was mainly centered on demonstrating various pharmacological properties of a different plant parts or of the whole extract itself (Vassallo, 2008; NewmanDj, 2016; Mir et al., 2021a). Presently, with the emergence of nutraceuticals, the applications of bioactive phytocompounds are not just limited to the consumption of natural products as dietary supplements, but to explore their specific therapeutic properties to develop potential drugs. With the vast diversity of bioresources, we have various plants, herbs, fruits, vegetables, seafood, meat, dairy products, nuts, berries, etc., are all rich with various phytocompounds which have neuroprotective properties are shown in Figure 4.

## Anti-Amyloid Effects

The key domain of investigation in AD pathophysiology is treatment techniques targeting Aβ oligomers. Various treatment techniques are employed, including blocking Aβ production (Silva et al., 2014) to reduce Aβ oligomers, lowering soluble levels of Aβ, and eliminating Aβ from the brain (Yang et al., 2016). AD causes neuroinflammatory reactions, mitochondrial malfunction, oxidative stress, loss of synaptic plasticity, transportation, and tau hyperphosphorylation, in addition to other associated cellular abnormalities. Aβ is produced as a predictable consequence of these physiological modifications (Tariq et al., 2021). Numerous botanical herbs demonstrated anti-amyloidogenic properties, indicating its influences on the accumulation and instability of pre-existing Aβ fibrillary aggregates in the CNS (Maccioni et al., 2010; Mir et al., 2021b; Nomoto et al., 2021; Wainwright et al., 2022). Aside from the medicinal herbs listed, studies have investigated the anti-amyloidogenic properties of ellagic acids, garlic acid, dry ginger extract, isolates of mulberry leaf, and caper buds, which are all consumable dietary elements (Fujiwara et al., 2006, 2009; Papandreou et al., 2006).

## Inhibitors of β and γ-Secretase

Targeting BACE-1 or γ-secretase enzymes is identified as the most effective treatment method for AD because Aβ build-up leads to aberrant enzymatic cleavage of APP by β- and γ-secretase enzymes, resulting in the production of Aβ oligomers (Durairajan et al., 2008; Fujiwara et al., 2009). Several herbal extractions that combine with β-secretases impact Aβ synthesis. Ellagic acid and punicalagin were reported to suppress β-secretase in, *Punica granatum L. (Lythraceae)*. Lipophilic alkylated flavonoids from *S. flavescens* Aiton *(Fabaceae)* have a strong non-competitive BACE-1 inhibition effect. Polyphenols derived from green and black tea, as well as *Smilax china L. (Smilacaceae)*, are effective BACE-1 inhibitors, which aids in slowing the advancement of Alzheimer’s disease.

## Tau Hypophosphorylation

Tau peptides action to stabilize microtubules, but their aberrant hyperphosphorylation causes tau agglomeration. AD is caused by these aggregates and constraining the development of tau clusters, controlling tau with kinases, limiting tau disintegration with chaperones, and stabilizing tau microtubules are successful methods to address post-symptomatic AD. Anti-tau effects have been demonstrated in herbal medications and extracts. Curcumin, a diarylheptanoid identified in turmeric (*Curcuma longa*) extracts, stimulates the generation of the anti-inflammatory IL-4 cytokine and lowers Aβ and tau concentrations in mice with Aβ overexpression (Kang et al., 2011).

The cinnamon (*Cinnamomum zeylanicum*) extraction inhibited tau assembly and additionally exhibited the inhibitory effect that is attributed to both cinnamaldehyde and procyanidin. The bioactive constituent paclitaxel, derived from *Taxus brevifolia*, exhibited therapeutic properties by combatting the functional loss in the tau pathology (Kang et al., 2011).

Severe oxidative damage in AD pathogenesis stimulates the production of reactive oxygen species (ROS) by activating microglial cells with Aβ oligomers (Syad and Devi, 2014). For instance, the validated efficacy of Ginko biloba contained metabolites such as, tocopherol, bioflavonoid pycnogenol, ascorbyl palmitate and other antioxidants ineffectively suppressing the apoptotic cells in the hippocampus of ApoE-deficient mice. Similarly, *Salvia officinalis* is notable for its antioxidant properties, anti-inflammatory characteristics, and mild suppression of AChEs (Fujiwara et al., 2009). The active element in sage, rosmarinic acid, inhibits the development of ROS, peroxidation of lipids, activation of caspase-3, fragmentation of nucleic acids, and Aβ oligomeric hyperphosphorylation of tau protein (Fujiwara et al., 2009). *Panchagavya Ghrita*, an Ayurveda composition, reduces seizures, cognitive decline, and oxidative damage (Joshi et al., 2015). As discussed in this review there are ample number of natural products that are neuroprotective in nature. The neuroprotective properties of some common fruits, vegetables, herb, berries, nuts species, and condiments are discussed in the proceeding section.

### Vaccinium angustifolium

The blueberry is high in anthocyanins, which are cytoprotective polyphenols having anti-oxidant properties. Blueberries aids in recovering cognitive and memory deficits in the CNS. For instance, Blueberry nutrition has been linked to improved cognition and motor function in elderly animals in preclinical investigations (Veerendra Kumar and Gupta, 2003; Calcul et al., 2012; Mathew and Subramanian, 2014). During a 12-week administration of natural blueberry juice, older persons with initial memory impairment reported significant amelioration in memory and cognitive function (Iuvone et al., 2006). This study discovered increased pair associated memorization and word identification recall. For instance, irradiated mice exhibited functioning benefits with blueberry supplementation in latency assessments, which entails retrograde training. The striatum is affected by polyphenols present in blueberry; it is important to note that this striatum is critical in retrograde learning (Youdim et al., 2000; Kumar et al., 2011). With the supplementation of blueberries increased accumulation of anthocyanin in the hippocampal and neocortex was noticed (Casadesus et al., 2004).

### Morus alba

Mulberries are enriched with hydroxyl stilbene, an antioxidant that is comparable to resveratrol but lacks an additional OH group that acts as a proton donor (Krikorian et al., 2010). In Aβ-induced neurotoxic conditions in cortical neurons, hydroxyl stilbene was found to exhibit neuroprotective properties. Hydroxyl stilbene reduces intracellular calcium ion levels, ROS production, and neurotransmitter glutamate production in Aβ-induced neurotoxicity (Shukitt-Hale et al., 2007). More research is required to determine the therapeutic potential of hydroxyl stilbene found in mulberry in AD models (Andres-Lacueva et al., 2005).

### Malus domestica

Apple Juice Concentrate (AJC) increased cognition, oxidative stress, and synaptic signaling in laboratory experiments (Spangler et al., 2003; Essa et al., 2012). In cultured cells research, Aβ and presenilin-1 concentrations were reduced, but synaptic transmission and ACh levels were boosted. The neuroprotective benefits of AJC are due to phytonutrients to antioxidant activity. PS-1 amplification is caused by faulty DNA methylation caused by a lack of SAM (S-adenosylmethionine). According to investigations, AJC offers adequate SAM to reduce the production of presenilin-1. Furthermore, mice administered with AJC had higher amounts of ACh in brain homogenates. As a result, drinking AJC is a healthy way to impede AD symptoms (Andres-Lacueva et al., 2005; Chen C. et al., 2021).

### Juglans regia

Walnut contains healthy triglycerides, α tocopherol, vitamins, and polyphenolic compounds, particularly ellagic acid. Research suggests that adapting to walnut-rich nutrition is linked to a decreased risk of neurological illnesses (Freyssin et al., 2020). Thioflavin T experiments were used to examine the possible suppressive properties of walnut extraction on Aβ fibril production (Remington et al., 2010). The amyloidogenic action of walnuts is due to phenolic acids. The findings imply that walnut extracts protect cells from death induced by Aβ. This beneficial effect is achieved by reducing the production of ROS and reducing cell membrane damage and DNA fragmentation. This beneficial impact could be due to the antioxidant polyphenols found in walnuts (Freyssin et al., 2020). As a result, walnut-rich nutrition is viable to prevent and delay the development of Alzheimer’s disease (Tripathi and Mazumder, 2020).

### *Piper nigrum* and *Piper longum*

Piperine is a nitrogen-containing alkaloid found in the fruit black pepper and long pepper. It is utilized in herbal treatment for therapeutic applications to manage a variety of diseases (Chan and Shea, 2006; Ichwan et al., 2021). Multimodal health advantages such as anti-depressive impact, better cognitive functioning, neuroprotective effect, and antioxidant properties activity has been documented based on pharmaceutical data (Chauhan et al., 2004; Muthaiyah et al., 2011; Hussain et al., 2021). It also has anti-inflammatory, anti-convulsant, analgesic, and anti-ulcerous properties. In AD patients, ethyl choline aziridinium can cause a cholinergic action. More research on the molecular and cellular mechanisms of neurogenesis is required. Piperine demonstrated significant enhancement in cognition and neurodegeneration in the hippocampus (Bano et al., 1987; Wattanathorn et al., 2008; Luca et al., 2021).

### Cinnamomum verum

Cinnamon is traditionally utilized as a condiment and medicinal supplement to treat various illnesses (Selvendiran et al., 2003). The alkaloids, flavonoids, ad cinnamic acid derivatives like cinnamaldehyde, eugenol, cinnamyl acetate, and cinnamyl alcohol, have versatile therapeutic applications like anti-inflammatory, antibacterial and antioxidant properties (Khan et al., 2021). The isolates prevent oligomers and amyloid filament production in fly and mouse models of AD. Cinnamon has a number of phytoconstituents that can penetrate the blood-brain barrier. Additional exploration of the impact of cinnamon extracts on various processes associated with AD is required (Hua et al., 2019).

### Allium sativum

*Allium sativum* is utilized in cuisine and healing all over the globe (D’Hooge et al., 1996). Tg2576 mice were used to examine the impact of aged garlic extraction. The use of aged garlic extract improved hippocampal-based cognitive dysfunction significantly. Supplemental research is needed to better understand the neuroprotective pathways (Bai and Xu, 2000).

### Zingiber officinale

*Zingiber officinale* is a common spice with ethnomedicinal characteristics similar to garlic. It’s regularly utilized as an infusion in ginger tea, or as nutritional supplements. The predominant bioactive constituents in ginger are gingerols, shagols, bisabolene, zingiberene, and monoterpenes (Singh et al., 2021). White and red ginger were tested for their ability to inactivate AChE and were measured using colorimetric analysis (Momtaz et al., 2018). Ginger extract has an inhibitory potential on AChE, particularly white ginger which displays a strong impact. Ginger’s suppressive properties work both collaboratively and independently (Frydman-Marom et al., 2011). Ginger’s ability to decrease oxidative damage is also useful in the prevention of AD (Tesfaye, 2021).

### Curcuma longa

Traditional therapies have widely used curcumin to treat various ailments. Though they have been mainly postulated to treat inflammation and dermal conditions, their neuroprotective protective properties have guided the researchers to explore the benefits of their derivatives in brain disorders (Chauhan and Sandoval, 2007). The anti-amyloidogenic ability, suppression of APP, and the inhibition of amyloid-β peptide of the curcumin derivatives is due to the presence of phenyl methoxy groups (Joshi et al., 2021). Curcumin exhibits anti-inflammatory, antioxidant, and anti-Alzheimer’s effects. Curcumin potentially inhibits AD-related enzymes like AChE, BChE, BACE-1, and aggregation of Aβ -tau proteins. Curcumins are beneficial in reducing oxidative stress (Joshi et al., 2021). Curcumin interacts with amyloid-beta and suppresses Aβ-tau agglomeration, and disintegrates the fibrils via meta binding which reduces the rate of nerve cell damage (Zhang M. et al., 2021). The generation of ROS is a critical factor in the pathogenesis of Alzheimer’s disease, which can be reduced by the antioxidant and free radical scavenging properties of curcumin (Talebi et al., 2021a). It lowers amyloid formation and oxidative stress-induced neuronal damage by inhibiting lipid peroxidation. Curcumin reduces protein oxidation and isopropyl propionate in the body (Talebi et al., 2021a).

### Cocos nucifera

The hypoglucose metabolism that occurs in the brain is a major indicator of Alzheimer’s. The lack of glucose supplementation to the brain has to compensate by an external source to which coconut oil can be a potential candidate as it is rich with medium-chain fatty acids which can directly reach the hepatic system (Oboh et al., 2012). The lack of cholesterol level in AD brain is an indicator of the disease. Therefore, while devising a treatment and management plan, it is important to include sufficient levels of saturated fats to maintain the levels of high-density lipoproteins. The saturated fats in the coconut oil supplement the brain with medium-chain triglycerides which are further converted into ketone bodies during periods of starvation or fasting (Bhat et al., 2019). The glutamate levels in the hippocampal and prefrontal cortex cells were significantly reduced in the rats administered with virgin coconut oil (Chainoglou and Hadjipavlou-Litina, 2020). The virgin coconut oil exhibits anti-oxidant, anti-inflammatory, and neuroprotective properties (Zhang L. et al., 2021).

### Bacopa monnieri

*Bacopa monnieri* is a nootropic herb that is rich in polyphenolic compounds like bacosides which prevents the brain from oxidative injury and age-related cognitive decline. The neuroprotective properties of the bacosides include destabilizing fibrils, suppression of Aβ-tau agglomeration, and protection from Aβ induced toxicity. The bacosides can easily cross the blood-brain barrier and they associate with the neurotransmitters to enhance memory, learning, and other cognitive functions. The administration of *B. monnieri* suppresses lipid peroxidation (Talebi et al., 2021b). The extract of *B. monnieri* reduced amyloid peptide-induced cell death by reducing the activity of AChE (Kappally et al., 2015). *B. monnieri* supplementation colchicine-induced dementia (Chatterjee et al., 2020).

### Elettaria cardamomum

*Elettaria cardamomum* has anti-bacterial, anti-microbial, anti-inflammatory, antioxidant properties which makes it a potential therapeutic compound (Alghamdi, 2018; Mirzaei et al., 2018). The cardamom oil has AChE inhibitory activity, anti-anxiety, and anti-depressant properties. A research study has revealed that a terpenoid isolated from *E. cardamomum* called alpha-terpinyl acetate which can be used as a suitable lead to develop a molecule that might have multi-targeted directed ligand (MTDL) potential and disease amelioration effects in AD (Abdul Manap et al., 2019). One more research study concluded that terpenoid-rich *E. cardamomum* extract prevents Alzheimer-like alterations induced in diabetic rats via inhibition of GSK3β activity, oxidative stress and pro-inflammatory cytokines (Uabundit et al., 2010).

### Salvia rosmarinus

Rosemary is an important herb in the Mediterranean diet and it has various culinary and therapeutic benefits. The herb is rich in antioxidants; especially phenolic diterpenes. This herb has anti-diabetic, anti-tumor, anti-inflammatory, antioxidant, neuroprotective properties, etc. Rosemary essential oil is rich in bioactive phytocompounds like terpineol, 1,8-cineole, pinene, camphene, and borneol. It also has an abundance of secondary metabolites, flavonoids, and phenolic acid derivatives like homoplantaginin, rosmarinic acid, gallocatechin, luteolin, etc. The diterpenes prevent the cells from oxidative damage and inhibit lipid peroxidation (Saini et al., 2019). The carnosic acid present in rosemary protects nerve cells from ischemic injury by generating the quinone derivatives which are accompanied by the loss of hydrogen radicals from their phenolic groups (Kumar and Kumari, 2021). The derivative of rosemary plays an important role in Aβ mechanism as they modulate amyloidogenic and non-amyloidogenic pathways; the major pathways associated with AD pathogenesis (Sengottuvelu, 2011). Carnosic acid reduces the generation of amyloid-β 1-42, Aβ-tau agglomeration and protects the cells from beta-amyloid-induced toxicity (Kumar and Kumari, 2021). Rosemary leaf extract enhances memory and learning ability and is directly proportional to the activity of enzymes like AChE, BuChE, etc., (Sengottuvelu, 2011).

### Crocus sativus

The extensively used spice saffron is rich in volatile compounds wherein safranal is the most abundant non-volatile compound of saffron. Non-volatile compounds like crocins, crocetin, quercetin and kaempferol, isophorones, carotenoids, zeaxanthin, lycopene, etc., are present in saffron (Gomaa et al., 2019; Chowdhury and Kumar, 2020). Saffron and its bioactive phytocompounds are speculated to have an effect on AChE activity, dopamine pathways signaling, Aβ peptides and tau aggregate formation, ROS, activation of glial cells, notch pathway, Keap1/Nrf2 signaling pathway, mitogen-activated protein kinases signaling pathway, etc. Saffron has been reported to prevent abnormal indicators such as cognitive performance, cognitive function, motor dysfunction, tremors, spasm and convulsions. Therefore, saffron has been postulated to treat various brain-related disorders; including Alzheimer’s (Gomaa et al., 2019; Chowdhury and Kumar, 2020).

### Camellia sinensis

Green tea’s catechin polyphenols help to slow down age-related cognitive deficits, motor nerves, and other associated neurological dysfunction in neurodegenerative disorders. Animal experiments with green tea extract showed a positive influence on brain and cognitive abilities. The catechin components can reverse neuropathological changes, stimulate nerve cell regeneration, and neuroplasticity. Catechin polyphenols play a role in the stimulation of antioxidative defense enzymes and in the prevention of monoamine oxidase and nitric oxide synthase (Wijeratne and Cuppett, 2007). Catechins regulate the activity of iron regulatory proteins, APP, AChE, and BuChE activity, fibrils disintegration, etc., (Wijeratne and Cuppett, 2007; Yoshida et al., 2014). The amyloid-induced dysfunction of mitochondria can be addressed with epigallocatechin-3-gallate and luteolin which can effectively restore the functions of the mitochondrial cells (Ozarowski et al., 2013). The treatment with epigallocatechin-3-gallate was effective in restoring mitochondrial function, generation of reactive oxygen species, and production of ATP in the hippocampus, cortex, and striatum mitochondria for up to 85% (Ozarowski et al., 2013).

### Moringa oleifera

The leaves of *Moringa oleifera* have anti-inflammatory, anti-oxidant and neuroprotective properties. *M. oleifera* was investigated on hyperhomocysteinemia-induced Alzheimer’s pathophysiology in mice. The study followed a 14-day homocysteine administration to establish AD-like pathology. *M. oleifera* shields cells against oxidative damage and cognitive deficits caused by Hcy administration. *M. oleifera* reduced dementia by restoring depleted synapse peptides like PSD93, PSD95, Synapsin 1, and Synaptophysin. It inhibited Hyc-induced tau hyperphosphorylation at multiple locations, including S-199, T-231, S-396, and S-404, while also lowering Aβ synthesis via BACE1 suppression (Shafiee et al., 2018). The leaf extraction of *M. oleifera* induces the differentiation of neurites and neuronal cell development, formation of spatial cognition and protects the cells from neurotoxicity (Finley and Gao, 2017).

### Punica granatum

In PC12 cells, *Punica granatum* extract was evaluated for its ability to protect cells from oxidative cytotoxicity. The findings of this investigation revealed that the ethyl alcohol extract of *P. granatum* reduced hydrogen peroxide instigated oxidative damage in PC12 cells (Mandel and Bh Youdim, 2012). *P. granatum* contains potent anti-dementia molecules like ellagic acid and punicalagin, which are BACE1 inhibitors. α -secretase, chymotrypsin, trypsin, and elastase were barely inhibited by ellagic acid and punicalagin, suggesting their specificity as inhibitors of BACE1 (Ide et al., 2018). The age-induced or scopolamine-induced retention impairments in mice dramatically improved after chronic administration of *P. granatum* extract and ascorbic acid for 3 weeks (Dragicevic et al., 2011). The age-induced or scopolamine-induced retention impairments in mice were dramatically improved after chronic administration of *P. granatum* extract and ascorbic acid for 3 weeks (Dragicevic et al., 2011).

### Rosmarinus officinalis

A polyphenol herbal ingredient called rosmarinic acid isolated from *Rosmarinus officinalis* is used to investigate the novel mechanism. It prevents the build-up of amyloid β (Aβ) in mice. Using DNA microarray analysis, the brain of mice (Alzheimer’s disease model) was examined to see if the dopamine signaling pathway was increased in the control group vs. those administered rosmarinic acid. Monoamines such as 3,4 dihydroxyphenyl acetic acid, levodopa, dopamine, and norepinephrine were increased in the cerebral cortex following rosmarinic acid administration. As a result, the ventral tegmental region and substantia nigra showed decreased expression of DA-degrading enzymes such as monoamine oxidase B. Monoamines have been shown to suppress amyloid aggregation by *in vitro* studies. *In vivo* studies showed that rosmarinic acid consumption increased monoamine concentrations via a decrease in monoamines B gene expression. According to this investigation, the increase in monoamines in the brain caused by rosmarinic acid may have a favorable effect on Alzheimer’s disease (Mahaman et al., 2018).

### Clitoria ternatea

Shankhpushpi is the popular name for it. A research study investigated the ethanolic root extract of *C. ternatea* against stress-induced amnesia in rats using the oral mode of administration at dosages of 150 and 300 mg/kg. Significant inhibitions of nitric oxide and DPPH production were detected in this experiment, as well as the protective effects of *C. ternatea* 77. Another research study examined the memory and central cholinergic activity of an alcoholic extract of *C. ternatea* roots and aerial portion in rats given 300 and 500 mg/kg. This extract boosted the activity of the enzyme acetylcholinesterase and the amount of acetylcholine in rat brains as well as memory function. *C. ternatea* root extract was shown to be more effective than aerial components (Kou et al., 2018).

### Melissa officinalis

The memory-enhancing action of *M. officinalis* extract was investigated via the cholinergic system. *M. officinalis* leaves were extracted using the maceration process with an ethanol concentration of 80 percent. *M. officinalis* extract (alone) was given intraperitoneally with scopolamine at various levels (50–400 mg/kg) before to training in a Morris Water Maze. After training was completed, the acetylcholinesterase enzyme level was determined in the hippocampus. *M. officinalis* extract at a dosage of 200 mg/kg significantly improved naive rats’ memory and learning and may potentially mitigate the scopolamine-induced learning impairment. However, the extract had no dose-dependent impact, and dosages greater than 200 mg/kg had no effect on memory enhancement or reversal in naive rats. Both scopolamine-induced memory impairment and naïve rats demonstrated a decrease in AChE activity. The findings indicated that *M. officinalis* extract may enhance the extract’s cholinergic and memory functions. This trial demonstrated that *M. officinalis* possesses high therapeutic effectiveness in Alzheimer’s disease-related memory impairment (Choi et al., 2011).

### Emblica officinalis

It is commonly known as Amla. It has been revealed that the effects of a hydroalcoholic extract of the fruit of *E. officinalis* on cholinergic function and oxidative stress were investigated in scopolamine-induced amnesic rats administered via the intraperitoneal route at dosages of 150, 300, 450, and 600 mg/kg. Amnesic mice had considerable reversal of GSH, MDA, and AchE activity (Kwak et al., 2005). In another research study, the tannoid principle of *E. officinalis* restored cognitive impairments and increased amyloid pathogenesis in rats exposed to aluminum chloride. Parle et al. evaluated the memory-enhancing effect of *E. officinalis* at three different dosages of 50, 100, and 200 mg/kg orally against diazepam and scopolamine-induced amnesia for 15 days (Kumar et al., 2009). Total cholesterol levels decreased considerably, whereas AchE activity reversed as well. All trials demonstrated that *E. officinalis* may be a very useful medicinal herb for treating Alzheimer’s disease (Hase et al., 2019).

### Glycyrrhiza glabra

*Glycyrrhiza glabra*, also known as liquorice, is an ornamental plant. The aqueous extract of *G. glabra* was tested for learning and memory in rats utilizing the oral route of administration at four different doses: 75, 150, 225, and 300 mg/kg versus the diazepam-induced amnesic model in rats by using the oral route of administration for 6 weeks. All *G. glabra* aqueous extracts improved memory and learning capacities according to the findings 58. Another research study employed the oral mode of administration to assess the memory and learning activity of Glabridin rich extract (5 and 10 mg/kg) and aqueous extract of liquorice (400 mg/kg) against diazepam and scopolamine-induced amnesia in mice, with results showing improvements in memory and learning activities (Taranalli and Cheeramkuzhy, 2000).

### Myristica fragrans

In this study, the n-butanol fraction of *M. fragrans* was studied against a scopolamine-induced model of Alzheimer’s disease at doses of 100 and 200 mg/kg. The mice’s AchE activity, retention transfer latency, and thiobarbituric acid reactive substances (TBARS) level all went down because the lipid peroxidation process was stopped by the drug. It also showed that the levels of glutathione peroxidase, SOD, and catalase had risen (Ozarowski et al., 2016). Another research study by Cuong et al. (2014) examined the methanolic extract of seeds from *M. fragrans* for its ability to block cholinergic transmission. AchE activity was slowed down by this extract: As a possible treatment for AD (Golechha et al., 2012). There was another study done by Parle et al. (2004) that found that the n-hexane extract of seeds from *M. fragrans* was tested for memory improvement at the doses of 5, 10, and 20 mg/kg orally against the diazepam and scopolamine model (Kumar et al., 2009). They didn’t know how the extract of *M. fragrans* made the memory better, but they observed that the extract of *M. fragrans* enhanced the memory (Kumar et al., 2009). All of the tests showed that *M. fragrans* can help treat AD.

### Evolvulus alsinoides

The leaves of *E. alsinoides* were tested for Alzheimer’s disease, antioxidants, as well as diabetes using several extracts (n-hexane, ethyl acetate, aqueous, methanol, petroleum ether, and chloroform). The effectiveness of FRAP reduction, AchE blockade, -glucosidase, and -amylase was investigated in this experiment. The aqueous extract performed better than the other extracts, according to the findings (Parle et al., 2004). In another research study, the oral administration of an ethanolic extract of *E. alsinoides* was reported to protect the brains of scopolamine-induced amnesic mice at two different doses of 250 and 500 mg/kg. The results revealed that AchE inhibition was effective.

### Celastrus paniculatus

The anti-Alzheimer’s diseases and antioxidant effects of a methanolic extract of seeds and its other organic soluble component of *C. paniculatus* were studied. Total reactive oxygen species formation, authentic peroxynitrite (ONOO) activity, and AchE and butyrylcholinesterase (BchE) inhibition were all significantly inhibited by this extract. The results revealed that EtOAc extract has the most potential compared to others *C. paniculatus* extract (Justin Thenmozhi et al., 2016). The effects of *C. paniculatus* seed oil on an aluminum chloride-induced neurodegenerative model were examined. All animal’s latency was increased. All biochemical parameters were analyzed, and it was discovered that AchE was considerably inhibited, malondialdehyde (MDA) levels significantly rose, and superoxide dismutase (SOD) levels significantly decreased. These findings concluded that *C. paniculatus* possesses powerful anti-disease Alzheimer’s activity (Desai et al., 2012).

### Lepidium meyenii

It is commonly referred to as black maca. The memory impairment generated by ovariectomized mice was examined using an aqueous extract of *L. meyenii* administered orally at two different doses of 0.5 and 2.0 g/kg. Chemicals such as monoamine oxidase (MAO), acetylcholinesterase (AchE), and malondialdehyde (MDA) were measured at various levels. In this experiment, the levels of MAO and AchE were both inhibited but there were no variations found in MDA levels. According to these findings, *L. meyenii* has the potential to impair memory (Singh, 2019).

### Nardostachys jatamansi

In young and old mice, the ethanolic extract of *N. jatamansi* was tested for memory and learning via the oral route at 50, 100, and 200 mg/kg against scopolamine and diazepam induced amnesia. In both old and young mice, a dose of 200 mg/kg improved learning and memory and restored amnesia caused by diazepam and scopolamine. This investigation demonstrated that *N. jatamansi* might be effective in the treatment of Alzheimer’s disease (Cuong et al., 2014).

Another research study, mice were given 200 and 400 mg/kg of methanolic extract of *N. jatamansi* for memory and cognition deficits in a sleep deprivation scenario. In behavioral tests, this trial indicated a significant improvement in cognition and memory. In this study, it was found that the methanolic extract of *N. jatamansi* has a neuroprotective effect (Sundaramoorthy and Packiam, 2020). A Drosophila AD model was used to test the ethanolic extract of *N. jatamansi* against amyloid toxicity *in vitro* and *in vivo* study. In SH-SY5Y cells, the extract of this plant decreased amyloid-induced cell death, reduced glial cell populations, reduced ROS levels, and suppressed A42-induced cell death. According to these findings, *N. jatamansi* could be an important plant in the treatment of Alzheimer’s disease (Alama and Haque, 2011).

### Coriandrum sativum

The Apiaceae family includes *Coriandrum sativum* L., which is popularly known as dhanya (Raut et al., 2015). Flavonoids like quercetin 3-glucoronide and polyphenolics such as protocatechuic acid, glycitin, and caffeic acid are among the most abundant phytochemicals present in *C. sativum*. The flavonoid content in seeds was reported to be 12.6 quercetin equivalents/kg, and the polyphenolic content to be 12.2 gallic acid equivalents/kg (Raut et al., 2015). The *C. sativum* extract increased total protein concentration and CAT, SOD, and GSH enzyme levels in the experimental rat, as well as reducing the amount of brain infarct, calcium levels, and lipid peroxidation (LPO). *C. sativum* leaves were also found to reduce scopolamine and diazepam-induced memory impairments (Rubio et al., 2011). Also, the leaves have antioxidant properties. They can scavenge free radicals like DPPH, and they can stop lipoxygenase and phospholipid peroxidation, which helps to improve memory.

### Cissampelos pareira

In mice, the hydroalcoholic extract of C. *pareira* was tested for learning and memory boosting activities against aging and scopolamine-induced amnesia at three distinct doses of 100, 200, and 400 mg/kg administered orally. The activity of acetylcholinesterase was reduced by these extracts. Due to its antioxidant and anti-inflammatory effect, a dose of 400 mg/kg (p.o.) demonstrated a more significant improvement in learning and memory-enhancing activity. Therefore, *C. pareira* may have an important role in Alzheimer’s disease management, according to the findings (Joshi and Parle, 2006). Table 2 summarizes the medicinal plants having anti-Alzheimer’s disease properties.

**TABLE 2:** Anti- Alzheimer’s activity of medicinal plants.

Plant	Part used	Bioactive phytocompounds	Activities	References
*Salvia officinalis*	Leaf extract	Diterpenes, rosmarinic acid, carnosic acid, quercetin	AchE, BchE inhibitor	Mook-Jung et al., 2001
*Rosmarinus officinalis*	Whole plant	Rosmarinic acid	Inhibition of Aβ accumulation	Mahaman et al., 2018
*Melissa officinalis*	Leaf extract	Flavonoids	Anticholinesterase activity	Choi et al., 2011
*Ginkgo ginseng*	Powder extract	Alkaloids, flavonoids	Memory enhancement	Xiao et al., 2010
*Ficus racemosa*	Bark extract	Tannins, saponins	Anticholinesterase activity	Song et al., 2014
*Ficus carica*	Fruit extract	Flavonoids, phenolic compounds, vitamins	Antioxidant and immunostimulant activity	Zhao et al., 2012
*Tinospora cordifolia*	Leaf extract	Flavonoids	Memory enhancement	Gomes et al., 2018
*Lepidium meyenii*	Root extract	Proteins	Anticholinesterase and antioxidant activity	Singh, 2019
*Panax ginseng*	Whole plant extract	Ginsenosides	Memory enhancement	Ma et al., 2020
*Celastrus paniculatus*	Seed extract	Alkaloids, sesquiterpenes	AchE, BchE inhibitory activity and anti-oxidant	Desai et al., 2012
*Coriandrum sativum*	Seed and leaf extracts	Carbohydrates, proteins, vitamins, volatile oils	Neuroprotective activity	Dajas, 2012
*Cissampelos pareira*	Root and leaf extracts	Alkaloids	Inhibition of Aβ accumulation	Joshi and Parle, 2006
*Moringa oleifera*	Leaf extract	Proteins, fatty acid	Inhibition of Aβ accumulation	Smach et al., 2015
*Nardostachys jatamansi*	Root and leaf extracts	Sesquiterpenes	Inhibition of Aβ accumulation and anti-oxidant properties	Cuong et al., 2014
*Evolvulus alsinoides*	Leaf extract	Alkaloids and flavonoids	Anticholinesterase and antidiabetic activity	Wesnes et al., 2000
*Bacopa monnieri*	Flower and leaf extract	Alkaloids, bacoside-A, terpenoids	Anticholine esterase, antidementia, inhibition of Aβ accumulation	Kappally et al., 2015
*Glycyrrhiza glabra*	Root and leaf extract and glabridin	Glabridin, volatile oils	Antiamnesic	Taranalli and Cheeramkuzhy, 2000
*Myristica fragrans*	Seeds, fruits and leaf extract	Terpenes, flavonoids	Antioxidant, memory enhancement, ache inhibitor	Kumar et al., 2009
*Magnolia officinalis*	Fruits, leaf extract	Flavonoids, phenolics and anthocyanins	Antioxidant, anticholinesterase, neuroprotective	Ahmed et al., 2011
*Punica granatum*	Peel, seeds, and leaf extract	Flavonoids, phenolics and anthocyanins	Antioxidant, neuroprotective	Sumanth and Mamatha, 2014
*Rhodiola rosea*	Leaf and root extract	Phenols, flavonoids, alkaloids	Neuroprotective, antiapoptotic	Ahmed, 2021
*Withania somnifera*	Fruits, leaf extract	Alkaloids, saponins, steroidal lactone, withanamides A and B	Anticholinesterase, inhibition of Aβ	Lee et al., 2008
*Sargassum sagamianum*	Whole part	Plastoquinones, sargaquinoic acid and sargam chromenol	AchE and BchE inhibitor	Mani and Parle, 2009
*Ecklonia cava*	Whole part	Eckol, 6-6’-bieckol, 8.8-bieckol, dieckol, phlorofucofuroeckol-a	Aβ accumulation, bche, ache inhibitory activity	Nomoto et al., 2021

## Neuroprotective Biomolecules: Possible Role Against Alzheimer’s Diseases

**FIGURE 5:**
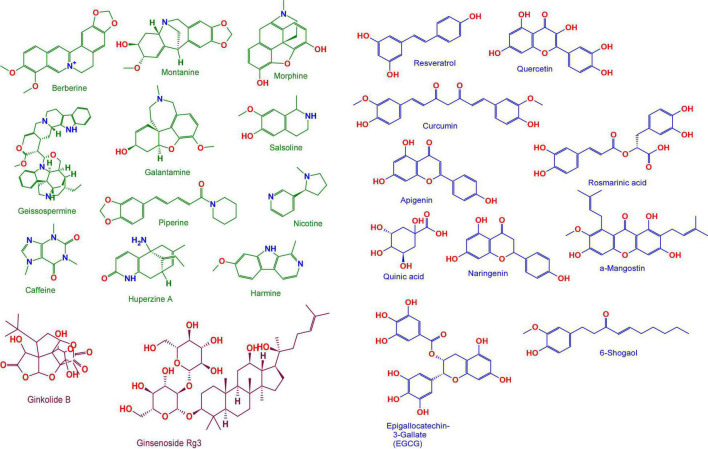
Anti-AD chemical compounds from medicinal plants. Berberine, Curcumin, Huperzine-A, Tetrandrine, Galantamine, Glaucocalyxin B, Oridonin, Quercetin, Curcumin, Naringenin, Resveratrol, Oxyresveratrol, Rosmarinic acid, Quinic acid, Apigenin, α-Mangostin, 6-Shogaol, Epigallocatechin-3-Gallate (EGCG), Ginkgolide B, Ginsenoside Rg3, Prosapogenin III and Diosgenin. Structures are obtained from the free chemical structure database (www.chemspider.com). For more details about their chemical properties see PubChem (http://pubchem.ncbi.nlm.nih.gov/).

**FIGURE 6:**
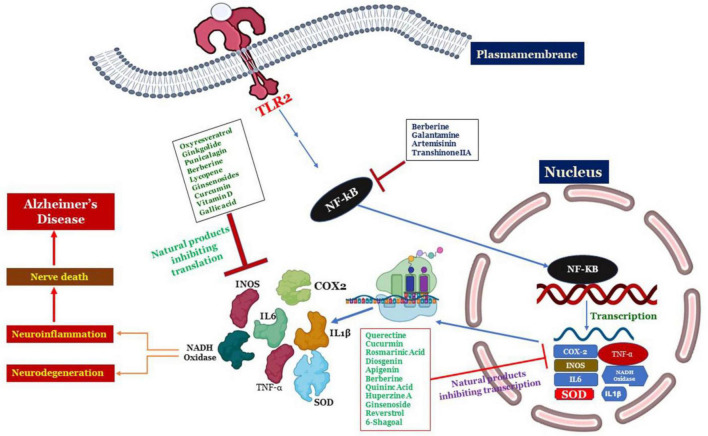
Schematics showing the activation of signaling pathways responsible for clinical features of AD through TLR signaling cascades largely governed by NK-KB resulting in the neuroinflammation and nerve degeneration. On the other hand the novel intervention of natural products such as Diosgenin, Prosapogenin III, Quercetin, Apigenin, Ginsenoside Rg3, Rosmarinic acid, Ginkgolide B, Limonoid, Quinic acid, Curcumin, Resveratrol, Berberine, 6- Shagoal, Ligraminol E4-O-β-d-xyloside, Huperzine A, Sophocarpidine, Naringenin, Epigallocatechin-3-galate (EGCG), Oxyresveratrol, α-Mangostin, Galantamine are shown to inhibit this signaling cascade at the junction of NK-KB, and then inhibition of transcription as well as translation of proteins responsible for neuroinflammation.

As discussed earlier a number of plant-derived or natural bioactive phytocompounds like phenols, alkaloids, strolls, carotenoids, flavonoids, etc., have cytoprotective, neuroprotective properties due to their antioxidant, anti-inflammatory, anti-apoptotic, properties. Origins and chemical structures of discussed phytochemicals were shown in Table 3 and Figure 5 and the mechanism of phytochemicals against AD is depicted in Figure 6.

**TABLE 3:** Plant-derived phytochemicals that affect Alzheimer’s diseases.

Phytochemicals	Plant source	Plant family	Pharmacological effects/Mechanism	References
Berberine	*Coptis chinensis*	Ranunculaceae	Activates the AKT/GSK-3/Nrf2 signaling pathways -mediated regulation, cholinergic activity-mediated neurite outgrowth, increases the release of NGF and BDNF, and suppresses the levels of Cox2, TNF-, NF-B, IL-1, and iNOS.	Sutalangka et al., 2013
Curcumin	*Curcuma longa*	Zingiberaceae	Activates PKC/ERK-dependent CREB regulation and AKT/GSK-3-dependent BDNF release, while inhibiting Cas3, TNF-, and NF-B levels.	Lee et al., 2012; Yadav et al., 2019
Huperzine-A	*Huperzia serrata*	Lycopodiaceae	Increase GST, SOD, and BDNF secretion Caspase-3, TNF-, NF-kB, and AChE inhibition	Fatima et al., 2017; Tang et al., 2017
Tetrandrine	*Stephania tetrandra*	Menispermaceae	Inhibits NF-KB and TNF- activity	Jayaprakasam et al., 2010
Galantamine	*Galanthus*	Amaryllidaceae	Inhibition of acetylcholinesterase, production of interleukin-IB, and microglial agglomeration	Choi et al., 2007
Glaucocalyxin B	*Rabdosia japonica*	Lamiaceae	Reduces the expression of nitric oxide, iNOS, and TNF- in LPS-activated microglial cells. Additionally, the stimulation of p38, NF-Kb, MAPK, and the formation of ROS were suppressed.	Jia et al., 2012
Oridonin	*Rabdosia rubescens*	Lamiaceae	In AD mice, activation of the BDNF and Nrf2 signaling pathways	Ambegaokar et al., 2003
Quercetin	*Morus alba*	Moraceae	Accumulation of hydroxyl radicals (OH) and superoxide anions (O2) Inhibitory action against LOX and COX enzymes	Wang et al., 2011
Curcumin	*Curcumin longa*	Zingiberaceae	Inhibition of the NF-KB pathway (PPARY) receptor activation	Wang et al., 2011
Naringenin	*Citrus paradise*	Rutaceae	Increases resistance to oxidative stress, cytokines, and NO, while decreasing NF-kB expression. In SH SY5Y cells, Nrf2 signaling is upregulated.	Shakibaei et al., 2007
Resveratrol	*Veratrum grandiflorum*	Liliaceae	Suppresses the expression of pro-inflammatory mediators such as NF-kB, TNF-, and IL-10 The decline in A42 and β-secretase 1 levels	Chen et al., 2006; Peng et al., 2006; Lei et al., 2015
Oxyresveratrol	*M. alba*	Moraceae	Reduce the release of NO from LPS-stimulated macrophages by decreasing the expression of the iNOS protein. TNF-, IL-1B, and IL-6 gene expression suppression	Chen et al., 1997; Woodruff-Pak et al., 2001
Rosmarinic acid	*Melissa officinalis*	Lamiaceae	By phosphorylating the ERK1/2 signaling pathway, increases cholinergic activity during cell differentiation. Interferes with fibrillization and β sheets	Gan et al., 2015; Wang et al., 2016
Quinic acid	*Pimpinella brachycarpa*	Apiaceae	Inhibits the production of a variety of inflammatory mediators in activated BV-2 microglial cell lines in response to LPS Protects SH-SY5Y cells against H2O2-induced harm through the activation of a variety of antioxidant enzymes	Raza et al., 2013; Sawda et al., 2017
Apigenin	*Passiflora edulis*	Passifloraceae	Apart from in PC 12 cells, inhibiting the synthesis of NO and PGE2. Reduced cytokine and NO oxide production	Chen et al., 2019
α -Mangostin	*Garcinia mangostana*	Clusiaceae	Prevent Aβ plaques from aggregating. Suppresses the β -secretase and – γ secretase enzymes, hence decreasing the synthesis of A.	Niidome et al., 2007
6-Shogaol	*Zingiber officinale*	Zingiberaceae	Increases NGF and pre- and postsynaptic proteins levels in the hippocampus COX-2, MAPK, and NF-KB repression	Wang C.P. et al., 2014; Moon et al., 2014
Epigallocatechin-3-Gallate (EGCG)	*Citrus sinensis*	Rutaceae	Blocking MAPK and NF-kB activation. Inhibit LPS-induced microglial activation	Bayat et al., 2012
Ginkgolide B	*Ginko biloba*	Ginkgoaceae	Inhibits pro-apoptotic protein expression and promotes NO production. Protective effect against neurotoxicity caused by reactive oxygen species	Yoo and Park, 2012
Ginsenoside Rg3	*Panax pseudoginseng*	Araliaceae	Reduced Aβ levels in the brains of mice with Alzheimer’s disease Suppressing the activation of the neurofibrillary tyrosine kinase (NF-kB)	Lee et al., 2013; Cornejo et al., 2017
Prosapogenin III	*Liriope platyphylla*	Asparagaceae	MAPK/NF-κB signaling inhibition. Phosphorylation of p38 is inhibited in response to H2O2-induced stress.	Soh et al., 2003
Diosgenin	*Dioscorea villosa*	Dioscoreaceae	Rectification of axonal degeneration. Enhancing memory deficits in the 5XFAD mouse model of Alzheimer’s disease COX-2, TNF-, and NF-κBp65 inhibition	Nikbakht et al., 2019

## Alkaloids

Besides intervening as muscarinic receptor agonists, anti-oxidants, anti-amyloid inhibitors, AChE and BuChE inhibitors, α-synuclein agglomeration inhibitors, dopaminergic and nicotine agonists, alkaloids help to alleviate the pathophysiology of AD (Rahman and Muralidharan, 2010).

Alkaloids have a broad spectrum of therapeutic potency in biomedicine, including analgesics (e.g., morphine), anti-diabetic (e.g., piperine), anti-tumor (e.g., berberine), and anti-microbial effects (e.g., berberine) (e.g., ciprofloxacin). Certain alkaloids have both stimulating and neuropsychiatric effects on the central nervous system (e.g., cocaine, caffeine, and nicotine) (e.g., psilocin). Despite the fact that alkaloids have a strong tradition and a wide range of properties, few are presented as functional and efficient medicines. They have a wide range of protective effects in conditions like seizures, psychiatric problems, cerebral ischemia, Alzheimer and memory lapses, anxiety, stress, and many more. Alkaloids suppress the establishment of neurodegenerative disorders by multiple mechanisms, including blocking the AChE, boosting GABA levels, and acting as NMDA antagonists (Rahman and Muralidharan, 2010; Liu et al., 2018).

## Terpenoids

Multiple research and clinical trials have validated that essential oils have positive benefits in AD patients. Plant essential oils and specific terpenes have been demonstrated to have antioxidant and AChEIs properties (Asgarpanah and Kazemivash, 2012). Effective anti-AD compounds include terpenoids such as ginsenosides, ginkgolides, and cannabinoids. Ginsenoside Rg3 (minimizes Aβ production by 84 percent in CHO-2B7 cells and by 31 percent in Tg2576) transgenic mice (Kulkarni et al., 2011; Avneet et al., 2018). Ginsenoside Rg3 lowers Aβ concentration by boosting Aβ breakdown and by increasing the production of neprilysin, a rate-limiting enzyme in Aβ degradation. PC12 cells are protected from Aβ-induced neurotoxic effects by ginsenoside Re. Moreover, ginsenoside Rb1 reduces neuroinflammatory biomarkers in the hippocampal cells, by reversing Aβ-induced cognitive impairment in mice. By boosting synapse plasticity in the brain, ginsenoside Rb1 has a positive influence on spatial working memory (Ng et al., 2015; Lima and Hamerski, 2019). Ginkgolides is a labdane-form of cyclic diterpenes that are often extracted from *Ginkgo biloba*. Ginkgolide A and B therapy preserves nerve cells from synaptic injury as measured by synaptophysin loss, a presynaptic synaptic indicator, and enhances nerve cell survival despite A-induced toxicity. Ginkgolide B protects hippocampus nerve cells against Aβ-directed cell death by boosting the synthesis of brain-derived neurotrophic factors and by reducing nerve cell apoptosis in hemorrhaging rat brain cells (Yoo and Park, 2012).

## Phenols

Resveratrol is proven to suppress the expression of pro-inflammatory molecules such as NF-kB and TNF-α in glial cells, while also increasing the amount of the anti-inflammatory cytokine IL-10, which is linked to Alzheimer’s diseases. Resveratrol improves spatial cognitive performance in Alzheimer’s disease rats via increasing anti-oxidant function. Resveratrol aids in the expression of SIRT1, which increases the preservation of nerve cells against ROS, free radicals, and Aβ -generated inflammation of the nerve cells (Chen et al., 2006; Awasthi et al., 2018). Oxyresveratrol, a compound derived from the *Morus alba* tree, reduces the production of the iNOS molecule in LPS- mediated macrophages, hence inhibiting the NO generation. Moreover, Oxyresveratrol has neuroprotective properties against Aβ protein-mediated neurotoxic effects in the cortical nerve cells, and anti-inflammatory and anti-apoptotic properties by lowering TNF-α, IL-1β, and IL-6 secretion and inhibiting caspase-1 and NF-kB expression (Chen et al., 2006). For its antioxidant capacity, ROS (OH, superoxide anions) scavenging effects, transversal BBB quercetin has been shown to have neuroprotective properties. Quercetin’s neuroprotective properties are mostly demonstrated through the dysregulation of cytokines via (MAPK) signaling pathways and p13K/Akt networks. Quercetin is also documented for inhibiting the LOX and COX proteases, which are related to the process of eicosanoids and the induction of NF-kB (Wang et al., 2011).

## Conclusion and Future Perspectives

Alzheimer’s is a complex, slow-progressing neurological illness. Even though AD associated pathologies are not greatly explored, current findings approved several factors responsible for its clinical manifestations. Multiple treatment strategies are explored at different stages as potential medication therapeutic interventions to successfully combat and control AD. FDA anti-AD medications deliver symptomatic treatment but have their drawbacks and side effects like nausea, vomiting, dizziness, headache, loss of appetite, loss of weight, diarrhea, etc. As a result, innovative alternate treatment techniques utilizing herbal medications to address AD is needed. Proper intervention in accordance with diseases progression ameliorates disease management. The irreversible damage to the brain cells and involuted pathophysiological, events associated with AD have always emphasized the need for the development of novel drugs and therapeutics, which render better outcomes with fewer or no side effects. Natural compounds and their bioactive phytochemicals have been shown to have significant neuroprotective potential in the treatment and management of AD, with limited negative side effects. Significant pharmacological properties like neuroprotective, anti-oxidant, anti-inflammatory, anti-apoptotic, etc., demonstrated by phytonutrients like tannins, alkaloids, phenols, carotenoids can be inspected to devise potential drugs. The degenerative pathway connected with Alzheimer’s disease is thought to be complex, despite the fact that it is not entirely comprehended. For the diagnosis and intervention of AD, neuroprotective treatments encompassing several molecular pathways are crucial. In the development of anti-AD drugs, organic product combinations or preparations containing several active pharmacological ingredients having the potential to execute diverse neuroprotective pathways and restorative mechanisms are sought. Green therapy could play a significant role in precluding AD and in devising therapeutics for symptom and disease management with the establishment of QA (Quality Assurance) and QC (Quality Control) guidelines to ensure the development of a safe and effective novel neuroprotective drugs. Our review strongly backs up use of medicinal plants and phytoconstituents alone or in combination with other compounds for effective treatments against Alzheimer’s disease with lesser side effects as compared to currently available treatments.

## Author Contributions

MM designed and supervised the study, and made a substantial contribution to the concept of study, and revision of the manuscript thoroughly. BB and AA equally contributed to this work in the analysis and writing of the manuscript. MM, BB, RM, WM, FA, and BA performed interpretation, drew the figures and tables, and critical review and drafting of the manuscript. All authors listed have made a substantial, direct, and intellectual contribution to the work, and read and approved the final manuscript.

## Conflict of Interest

The authors declare that the research was conducted in the absence of any commercial or financial relationships that could be construed as a potential conflict of interest.

## Publisher’s Note

All claims expressed in this article are solely those of the authors and do not necessarily represent those of their affiliated organizations, or those of the publisher, the editors and the reviewers. Any product that may be evaluated in this article, or claim that may be made by its manufacturer, is not guaranteed or endorsed by the publisher.
